# Identification of major trauma using the simplified abbreviated injury scale to estimate the injury severity score: a diagnostic accuracy and validation study

**DOI:** 10.1186/s13049-025-01320-7

**Published:** 2025-01-29

**Authors:** David Eidenbenz, Tobias Gauss, Tobias Zingg, Vincent Darioli, Cécile Vallot, Pierre-Nicolas Carron, Pierre Bouzat, François-Xavier Ageron

**Affiliations:** 1 Department of Emergency Medicine, Lausanne University Hospital and University of Lausanne, 21 Rue du Bugnon, BH 09, 1011 Lausanne, Switzerland; 2 Department of Anesthesiology and Intensive Care Medicine, Grenoble Alps University Hospital, Grenoble, France; 3 Department of Visceral Surgery, Lausanne University Hospital, Lausanne, Switzerland; 4 Northern French Alps Emergency Network, Department of Public Health, Annecy Genevois Regional Hospital, Pringy, France

**Keywords:** Abbreviated injury scale, Injury severity score, Trauma, Registries, Major traumatic injury, Wounds and injuries

## Abstract

**Background:**

The Abbreviated Injury Scale (AIS) and Injury Severity Score (ISS) grade the severity of injuries and are useful for trauma audit and benchmarking. However, AIS coding is complex and requires specifically trained staff. A simple yet reliable scoring system is needed. The aim of this study was two-fold. First, to develop and validate a simplified AIS (sAIS) chart centred on the most frequent injuries for use by non-trained healthcare professionals. Second, to evaluate the diagnostic accuracy of the sAIS (index test) to calculate the simplified ISS (sISS) to identify major trauma, compared with the reference AIS (rAIS) to calculate the reference ISS (rISS).

**Methods:**

This retrospective study used data (2013–2014) from the Northern French Alps Trauma Registry to develop and internally validate the sAIS. External validation was performed with data from the Trauma Registry of Acute Care of Lausanne University Hospital, Switzerland (2019–2021). Both datasets comprised a random sample of 100 injured patients. Following the Standards for Reporting of Diagnostic Accuracy Studies 2015 guidelines, all patients completed the rAIS and the sAIS. The sISS and the rISS were calculated using the sAIS and the rAIS, respectively. Accuracy was evaluated with the mean difference between the sISS and the rISS and the Pearson correlation coefficient. A clinically relevant equivalence limit was set at ± 4 ISS points. Precision was analyzed using Bland-Altmann plots with 95% limits of agreement.

**Results:**

Accuracy was good. The mean ISS difference of 0.97 (95% CI, −0.03 to 1.97) in the internal validation dataset and − 1.77 (95% CI, − 3.04 to 0.50) in the external validation dataset remained within the equivalence limit. The Pearson correlation coefficient was 0.93 in the internal validation dataset (95% CI, 0.90–0.95) and 0.82 in the external validation dataset (95% CI, 0.75–0.88). The limits of agreement were wider than the predetermined relevant range.

**Conclusions:**

The sAIS is accurate, but slightly imprecise in calculating the ISS. The development of this scale increases the possibilities to use a scoring system for severely injured patients in settings with a reduced availability of the AIS.

*Trial registration*: Retrospectively registered.

**Supplementary Information:**

The online version contains supplementary material available at 10.1186/s13049-025-01320-7.

## Background

The organization of care in trauma systems has been shown to reduce mortality [1]. The evaluation of the quality and performance of trauma systems and centres is based on the analysis of data collected from trauma registries [2]. Scaling and scoring systems are needed to stratify baseline risk, assess severity of injury, and allow for interhospital comparison and benchmarking.

The Abbreviated Injury Scale (AIS) developed by the Association for the Advancement of Automotive Medicine (AAAM) is a standardized scale describing the severity of injuries of the entire body [3]. It classifies each injury in nine predefined anatomical regions and by severity, ranging from 1 (minor injury) to 6 (maximal injury). The 2008 AIS contains 1999 injury descriptors and the 2015 AIS contains 2006 injury descriptors. The AIS is used for the calculation of the Injury Severity Score (ISS), which assesses the overall severity in injured patients. The ISS is the sum of the square of the highest AIS severity code in the three most severely-injured body regions and is used to define major trauma (usually an ISS ≥ 16) and to retrospectively characterize the case-mix of a trauma centre or system and their outcomes [4].

One of the main limitations of AIS coding is its complexity and cost of use. Coding requires specifically trained and accredited staff and coders must follow a course of two days with prerequisites in basic anatomy and medical terminology. They must also obtain recertification every five years. This limits the availability of this trauma scoring system in general hospitals and resource-limited countries with a high incidence of trauma [5]. There is a need to simplify the burden of coding in trauma registries.

The aim of this study was two-fold. First, to simplify the AIS classification into a condensed chart for ISS calculation and to internally and externally validate this simplified version for use by non-trained healthcare professionals. Second, we aimed to evaluate the diagnostic accuracy of the simplified AIS (sAIS) to calculate the simplified ISS (sISS) to identify major trauma.

## Methods

### Study design

This retrospective study was conducted in three steps. First, we developed the simplified scale (sAIS) using the 100 most frequent injuries collected in the Northern French Alps Trauma Registry (TRENAU). Second, for the internal validation, we examined the sAIS classification performance by randomly selecting 100 injured patients in the TRENAU Registry, which were rated by 10 French physicians. Third, we externally validated the sAIS by randomly selecting 100 injured patients included from a different dataset, the Trauma Registry of Acute Care (TRAC) of Lausanne University Hospital (Lausanne, Switzerland), which were rated by eight Swiss physicians and two research nurses. The study was conducted and reported in accordance with the Standards for Reporting Diagnostic Accuracy Studies (STARD) 2015 guidelines [6, 7].

### Study setting and participants

Two trauma registries from 14 trauma centres were used to randomly select study participants. We used data collected by the French TRENAU Registry between 1 January, 2013 and 31 December, 2014 [8] for the development and internal validation of the sAIS. The Registry includes two level I, one level II, and 10 level III trauma centres in an inclusive trauma system. External validation was completed using data from the TRAC collected from 1 June, 2019 to 1 June 1, 2021. The TRAC includes one level I trauma centre (Lausanne University Hospital) in an exclusive trauma system of the state of Vaud (Switzerland), regrouping seven general hospitals and one university hospital [9].

The two registries collected data following the Utstein template for the uniform reporting of data following major trauma [10]. The certified coder scoring the reference AIS (rAIS) and reference ISS (rISS) in the TRAC had six years of coding experience with 5,028 cases rated throughout her career. Another certified coder scored the rAIS in the TRENAU Registry. The AIS 2008 classification was used until 31 December, 2019 and the AIS 2015 since 1 January, 2020. Inclusion criteria were any suspected major trauma based on physiological, anatomical and anamnestic criteria. Exclusion criteria were patients with isolated burns (including electric injury), out-of-hospital traumatic cardiac arrest, asphyxia or hanging without other injuries, and drowning. The following data were extracted: rAIS; rISS; age; gender; type of trauma; mechanism of injury; heart rate; systolic blood pressure; Glasgow Coma Scale; and survival status at hospital discharge (alive or dead). Coders included in the study to score the sAIS and calculate the sISS were randomly selected among all physicians involved in trauma care in the emergency department (ED) or intensive care unit (ICU) in one trauma centre of each trauma system. Clinicians did not receive any previous training or certification in AIS coding.

### Development of the sAIS

We extracted the 100 traumatic injuries most frequently reported in the TRENAU Registry between 2013 and 2014 (Additional File 1), which represent 90% (in proportion of reporting) of all AIS diagnoses described in the registry. We classified the 100 diagnoses into six anatomical regions (head and neck, face, chest, abdomen and pelvis, extremities, external) and by severity from 1 (minor injury) to 6 (maximal injury) to develop the sAIS (Table 1). We checked if all organs and all types of injury (skeletal, vascular, neurological, internal organs) were represented in the classification. We ensured that every category of severity was represented for each organ. If not, we added a generic injury in the chart for the missing organ or missing type of injury (e.g., retina detachment) in order to cover all possible diagnoses. We grouped diagnostics in generic categories by severity to reduce the number of items of the condensed chart. The sAIS was designed to be used by non-trained healthcare professionals.

**Table 1:** simplified abbreviated injury scale

	1 Minor	2 Moderate	3 Severe non-vital	4 Severe vital	5 Critical
Head + neck	Cerebral concussion without loss of consciousness (headache or vertigo possible) Scalp abrasion, laceration, contusion Skin abrasion of the neck Cervical strain	Cerebral concussion with brief LOC (< 1 h) Skull vault fracture Petechial hemorrhage or subarachnoid hemorrhage without LOC Cervical spine fracture (vertebral body ≤ 20% loss of anterior height, spinous, transverse, facet, lamina, pedicle) Larynx, pharynx or trachea contusion	Concussion with loss of consciousness 1–6 h Penetrating injury to skull ≤ 2 cm Basilar skull fracture Cerebrum contusion / subarachnoid hemorrhage with LOC Cervical spine fracture of the vertebral body > 20% loss of anterior height Cervical cord contusion Larynx, trachea or pharynx perforation	Cerebral concussion with LOC 6-24 h Skull vault fracture, depressed > 2 cm Intracranial hematoma* (< 1 cm diameter) Cord contusion with incomplete neurological deficit Asphyxia with neurological deficit	Cerebral concussion with LOC > 24 h Skull penetrating injury > 2 cm Intracerebral hematoma** (> 1 cm diameter) Brainstem lesion Severe brain edema Cord contusion with quadriplegia or paraplegia with no sensation -Asphyxia with cardiac arrest
Face	Facial skin abrasion, contusion, laceration ≤ 10 cm Tongue laceration, Closed fracture (nose, mandible, zygoma) -Tooth dislocation, avulsion, fracture -Eye injury (cornea, sclera, uvea, vitrous, retina, foreign body Ear injury (ear canal, middle- or inner ear)	Skin abrasion, contusion, laceration > 10 cm Nose, mandible or zygoma open, displaced or complex fracture Orbit fracture LeFort I or II Fracture Eye avulsion (unilateral) Retina detachment Optic nerve avulsion	Lefort III fracture		
Chest	Rib fracture (1)* Thoracic wall or sternum contusion *add 1 point if a pneumothorax is associated with the rib fracture	Rib fractures (2)* Sternum fracture Lung contusion, unilateral (< 1 lobe) Thoracic skin laceration > 20 cm Pleura laceration Thoracic spine fracture (vertebral body ≤ 20% loss of anterior height, spinous, transverse, facet, lamina, pedicle)	Rib fractures (3–5) Hemo-pneumothorax Lung contusion or laceration, unilateral, ≥ 1 lobe Lung laceration, bilateral and < 1 lobe, or lung laceration unilateral ≥ 1 lobe Brachiocephalic, subclavian, pulmonary artery or vena cava inferior laceration/perforation (blood loss ≤ 20%) Heart laceration with hemopericardium, without perforation or tamponade Thoracic spine fracture of the vertebral body > 20% loss of anterior height Thoracic cord contusion with transient neurological signs	Flail chest Hemothorax (> 1000 ml) Pneumothorax (major, > 50% lung collapse or bilateral) Pulmonary contusion or laceration, bilat. (≥ 1 lobe) Intimal tear of the thoracic aorta Rupture or transsection of major vessels (subclavian, cava) Major contusion of the heart (LVEF < 25%) Tamponade Diaphragm rupture with herniation Esophagus, main stem bronchus or trachea perforation Cord contusion (thoracic) with incomplete neurological deficit (preservation of some sensation or motor function)	Bilateral flail chest Tension pneumothorax Rupture or transsection or disruption of the thoracic aorta and pulmonary artery (blood loss > 20%) Heart injury with perforation Rupture or transsection of the main stem bronchus, or trachea Cord contusion (thoracic) with complete cord syndrome (paraplegia, no sensation) Drowning with cardiac arrest
Abdomen	Abdominal, pelvic, perineal or lumbar contusion or abrasion	Abdominal skin laceration > 20 cm Stomach, duodenum, jejunum, ileum colon, rectum, anus, bladder, ureter, or urethra laceration/contusion Kidney, liver, pancreas or spleen minor contusion or capsular tear Retroperitoneum hemorrhage/hematoma Lumbar spine fracture (vertebral body loss ≤ 20% of anterior height, spinous, transverse, facet, lamina, pedicle)	Abdominal injury with blood loss > 20% -Stomach, duodenum, jejunum, ileum, colon, rectum, anus, bladder, ureter or urethra perforation Kidney, liver, pancreas or spleen major contusion or parenchymal laceration Arterial injury/laceration/perforation (celiac, iliac common-internal–external, mesenteric), venous injury (vena cava inf.) Lumbar spine fracture of the vertebral body > 20% loss of anterior height Lumbar cord contusion with transient neurological signs Cauda equina contusion (transient neurological signs or incomplete cauda equina syndrome)	Abdominal aorta laceration/perforation Stomach, duodenum, jejunum, ileum, colon, rectum rupture or transection Kidney, liver, extensive pancreas, or spleen major laceration (OIS IV) Arterial rupture/transsection (celiac, ilaca common-int.-ext.) venous rupture (vena cava inf.) Incomplete cord syndrome (lumbar) with preservation of some sensation or motor function Complete cauda equina syndrome	Abdominal aortic rupture Kidney, liver, pancreas or spleen disruption or complexe laceration (OIS V) Cord contusion (lumbar) with complete cord syndrome (paraplegia, no sensation)
Extremities + Pelvis	Various contusions Skin abrasions or lacerations (≤ 20 cm body, ≤ 10 cm hand) Tendon tear (upper extremity) Acromioclavicular joint, shoulder, elbow, wrist, hand, hip, knee, ankle or foot sprain Finger or toe dislocation/fracture Incomplete muscle injury *add 1 point if open fracture	Pelvic ring fracture, stable* (ischial tuberosity, pubic ramus, symphysis, not involving posterior arch) Shoulder, elbow, wrist, hip, knee, ankle dislocation Humerus*, clavicle, radius*, ulna*, carpus, metacarpus, tibia*, fibula*, calcaneus, talus, tarsal, or metatarsal fracture Axillary, brachial or popliteal artery minor laceration (blood loss ≤ 20%) Nerve injury Tendon tear or disruption (inferior extremity)	Amputation below elbow or knee Pelvic ring fracture, partially or vertically stable, with incomplete disruption of post. arch (open book sacroiliac joint disruption, symphysis pubis separation)* Femur fracture (proximal, shaft or distal), close or open Sciatic nerve laceration Axillary, brachial or popliteal artery laceration or rupture (blood loss > 20%) femoral (blood loss > 20%) Femoral artery injury (blood loss ≤ 20%) Degloving entire extremity, above elbow	Amputation above elbow or knee Pelvic ring fracture, unstable (vertical shear, pubic rami fracture, sacroiliac fracture/dislocation) Femoral artery rupture/transection (blood loss > 20%)	Bilateral amputation above elbow or knee Pelvic fracture partially stable or unstable, with blood loss > 20% or open
External	Superficial abrasion, contusion, hematoma or laceration Wounds ≤ 20 cm on the body Frostbite, stage 1 Burn: 1st degree, any Burn: 2nd or 3rd degree < 10% TBSA	Wounds > 20 cm body Deep frostbite Burn: 2nd or 3rd degree with 10–19% TBSA Electrical injury	Burn: 2nd or 3rd degree with 20–29% TBSA Electrical injury with muscle necrosis	Burn 2nd or 3rd degree with 30–39% TBSA	Burn 2nd or 3rd degree with 40–89% TBSA Electrical injury with cardiac arrest

AIS 6 is used for the following injuries specifically assigned to severity level 6:

Head crush injury with massive destruction of skull, brain and intracranial contents; brain stem transection, laceration, or destruction

Decapitation

Aorta thoracic injury with hemorrhage not confined to the mediastinum

Heart injury with multiple lacerations and ventricular rupture

Thoracic crush injury with massive bilateral destruction (skeletal, vascular, organ and tissue system)

Hepatic avulsion (total separation of all vascular attachments)

Burn: 2nd or 3rd degree with ≥ 90% TBSA

AIS 6 is not an arbitrary choice simply because a patient died. If one injury is coded AIS 6, the ISS is 75

*LOC* loss of consciousness, *TBSA* total body surface area, *OIS* organ injury scaling

^*^Extradural or subdural hematoma < 1 cm thick (≤ 50cc) or contusion or intracerebellar > 4 cm thick or midline shift > 5 cm

^**^Bilateral extradural or subdural hematoma or > 1 cm thick (> 50cc) extradural or subdural hematoma or contusion or important intracerebral hematoma (> 50cc)

### Internal validation

We internally validated the sAIS using data from the TRENAU Registry from 1 January, 2013 to 31 December, 2014. Eight physicians from the ED and two from the ICU of a level 1 trauma centre (Annecy-Genevois Hospital) were randomly chosen among the ED (n = 29) and ICU (n = 16) teams, without any previous experience in AIS coding. They were asked to independently calculate the simplified ISS (sISS) of 10 cases each using the sAIS (Table 1), reported by body region using a data collection sheet (Additionnal File 2), and blinded to the rISS reported in the trauma registry. The 100 patients were selected by stratified randomization according to the ISS severity. The physicians used the ED medical records and radiological reports (radiography, computed tomography, magnetic resonance imaging, ultrasound) to rate their 10 cases, if available. No cases were rated by more than one physician.

### External validation

We externally validated the sAIS using patient cases from the TRAC Registry. A similar process was used as previously detailed for the internal validation. Six registrar physicians, two senior consultants and two clinical research nurses from the ED were chosen among the team (n = 39) by randomization to calculate the sISS of 10 cases per participant, i.e., 100 patients in total.

### Reference and index diagnostic tests

The reference diagnostic test for major trauma identification was the rISS calculated using the rAIS and scored by a specifically trained and accredited coder. The index diagnostic test under evaluation was the sISS, calculated using the sAIS and scored by non-trained healthcare professionals.

### Outcome

The primary outcome was the accuracy of the sISS calculated using the sAIS compared with the rISS calculated using the rAIS ©2008 and ©2015.

### Statistical analysis

We present continuous data as means and standard deviation (SD) when normally distributed or medians and interquartile range (IQRs) when not normally distributed. We report categorical data as numbers and percentages. We used Student's t-test to compare continuous and normally distributed data and the Mann–Whitney test for continuous and non-normally distributed data. We defined a two-tailed *p*-value of < 0.05 as statistically significant.

First, we assessed the difference between the two methods at the whole trauma population level. We estimated the mean difference between the rISS and the sISS, which represents a measure of the accuracy. We considered a clinically relevant limit of equivalence of ± 4 ISS points for the bias. For a SD of the ISS of 9.5 and a limit of equivalence of 4 ISS points, 97 patients were required to ensure a power of 80% with a significance level of 5%. We estimated the Pearson correlation coefficient (r) as another measure of accuracy. The relationship between the sISS and the rISS was described by using scatterplots and local polynomial regression in a calibration plot.

Second, as a measure of the precision at an individual patient level and to examine the agreement between the sISS and the rISS, we used the Bland–Altman method to plot the bias and the limits of agreement (LoA). Assuming a normal distribution, the LoA represent the mean of the difference ± 2 SD of the difference. We considered a relevant LoA range of ± 9 ISS points. We used two different limits of ISS variation. For the precision at an individual level, we used a LoA range of ± 9 ISS points, corresponding to an increase in the severity of an injury from an AIS 4 to 5, as described by Ringdal et al. [11]. For the accuracy at the population level, we chose a narrower limit of equivalence of ± 4 ISS points as clinically relevant. In addition, as the ISS is used to classify major trauma (ISS ≥ 16), we assessed the agreement of major trauma classification by using the Cohen’s kappa statistic between the two methods [12]. We performed a complete case analysis as no missing values were reported.

As we suspected an imperfect gold standard bias, an independent trained coder reviewed the rISS of the patient cases of the external validation dataset when the difference between rISS and sISS was outside the calculated LoA limit. We performed a sensitivity analysis of the sISS compared with the corrected rISS. Analyses were performed with Stata version 16 (Stata Corporation, College Station, TX, USA).

## Results

The demographic and clinical characteristics of patients selected in each trauma registry are summarized in Table 2 (mean age, 41 [TRENAU] and 52 [TRAC] years). Main mechanisms of injury were as follows: low energy fall (n = 11 [11%] TRENAU; n = 31 [31%] TRAC); motor vehicle collision (n = 28 [28%] TRENAU; n = 10 [10%] TRAC); high energy fall (n = 27 [27%] TRENAU]; n = 10 [10%] TRAC); and motorcycle crash (n = 19 [19%] TRENAU; n = 12 [12%] TRAC]). The main anatomical regions injured were the head/neck, chest and lower limb/pelvis. The median [IQR] ISS was 18 [9–29] in the TRENAU and 13 [8–20.5] in the TRAC.

**Table 2:** Patient characteristics

	Internal validation dataset TRENAU (France) N = 100	External validation dataset TRAC (Switzerland) N = 100
Sex (male)	80	66
Age (years), mean (SD)	41 (19)	52 (22)
Penetrating injury	1	11
*Mechanism of injury*		
Motor vehicle collision	28	10
Motorcycle crash	19	12
Bike crash	6	19
Pedestrian hit by vehicle	1	3
High energy fall	27	10
Low energy fall	11	31
Gunshot—stabbing	1	11
Struck by	2	4
Heart rate [/min], mean (SD)	89 (22)	85 (23)
Systolic BP [mmHg], mean (SD)	124 (21)	139 (24)
GCS, median (IQR)	12 (5)	15 (2)
Death in the first 24 h	1	1
In-hospital death	7	4
*AIS* ≥ *3*		
Head or neck	28	42
Chest	38	22
Extremities or pelvic girdle	16	13
Abdominal or pelvic contents	17	7
Face	4	2
External	0	2
ISS, median (IQR)	18 [9–29]	13 [8–20.5]

Low energy fall: defined as a fall from a standing height or less than 3 m

*AIS* Abbreviated Injury Scale, *BP* blood pressure, *CI* confidence interval, *IQR* interquartile range, *ISS* injury severity score, *GCS* Glasgow coma scale, *SD* standard deviation

### Trauma population level

The mean of the difference between the rISS and the sISS was 0.97 (95% CI, − -0.03 to 1.97) in the internal validation dataset and − 1.77 (95% CI, − 3.04 to − 0.49) in the external validation dataset, which is included in the equivalence limit of ± 4 ISS points (Table 3). For 11 cases of the external validation dataset, the difference between the sISS and rISS was outside the calculated LoA. After recoding by an independent coder, the mean difference was − 0.86 (95% CI, − 1.87 to 0.15).

**Table 3:** Performance Indicators in the internal and external validation datasets

	Internal validation dataset TRENAU (France) 2013–2014	External validation Dataset TRAC (Switzerland) 2019–2021	External validation dataset TRAC (Switzerland) 2019–2021 (rISS corrected*)
Bias between rISS and sISS, mean [ISS points] (95% CI)	0.97 (− 0.03 to 1.97)	− 1.77 (− 3.04 to − 0.49)	− 0.86 (− 1.87 to 0.15)
Pearson correlation coefficient (95% CI)	0.93 (0.90 to 0.95)	0.82 (0.75 to 0.88)	0.89 (0.84 to 0.93)
Limit of agreement [ISS points]	− 9.1 to 11.1	− 14.6 to 11.0	− 11.1 to 9.4
Proportion of patients outside the limit of agreement (− 9 to + 9)	3%	11%	4%
% of agreement for ISS ≥ 16, (Cohen’s kappa)	89% (0.77)	81% (0.62)	85% (0.70)

*CI* confidence interval, *TRAC* Swiss Trauma Registry, *TRENAU* Northern French Alps Trauma Registry

^*^External validation dataset corrected for imperfect gold standard bias: calculation based on the rISS corrected

**Fig. 1:**
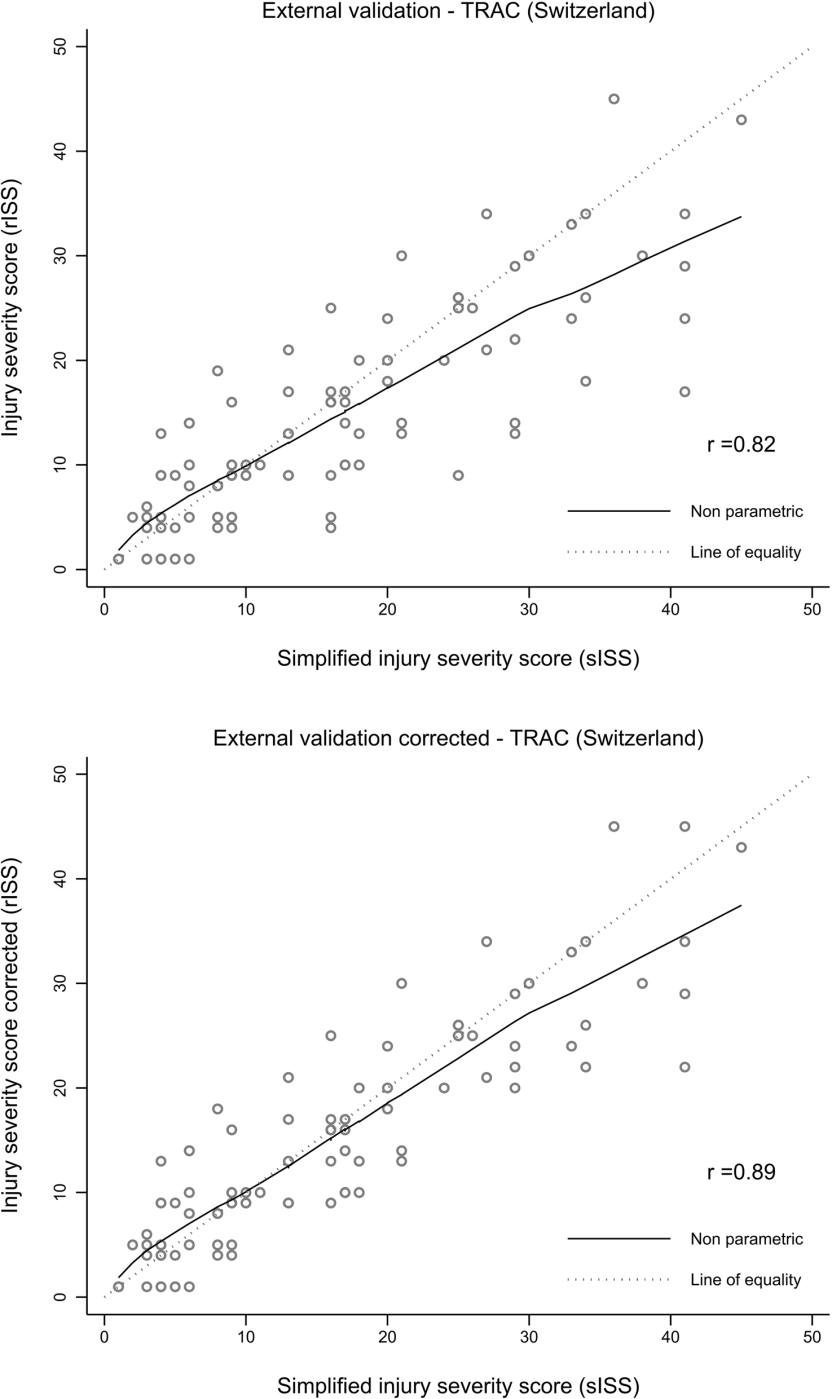
Calibration plot for the external validation dataset (uncorrected and corrected)

The Pearson correlation coefficient was 0.82 (95% CI, 0.75–0.88) in the external validation dataset and 0.89 (95% CI, 0.84–0.93) after correction. The sISS slightly underestimated a lower ISS (< 9) and overestimated a higher ISS (> 25) (Fig. 1).

### Individual patient level

**Fig. 2:**
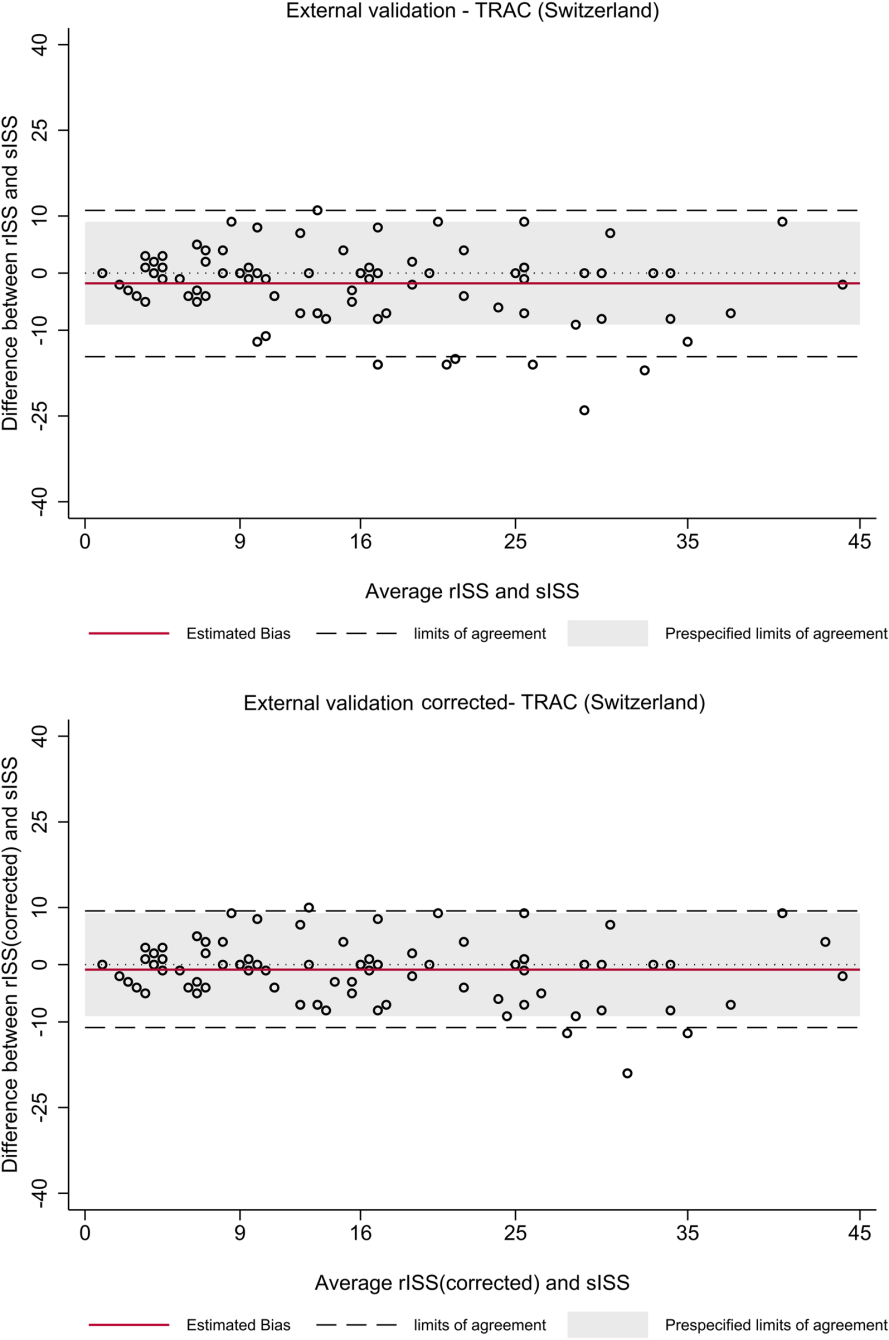
Bland-Altmann plot for the external validation dataset

The Bland–Altman plot showed a low estimated bias, but a LoA range slightly outside the predefined relevant range of ± 9 ISS points in the uncorrected external validation dataset (Fig. 2; Table 3). After correction for an imperfect gold standard bias, the LoA range was narrower (− 11.1 to 9.4), but remained outside the predefined relevant limits of agreement of ± 9 ISS points (Fig. 2). The proportion of patient cases outside the LoA was 11% in the external validation dataset and 4% in the corrected external validation dataset. Most outliers presented a higher ISS (V-shape in the Bland–Altman plot). The calibration plot and the Bland-Altmann plot for the internal validation dataset is presented in the Additional File 3.

## Discussion

We developed and validated an sAIS to calculate the ISS and were able to demonstrate an excellent accuracy with a low ISS difference. However, as values of the LoA were outside the predefined relevant range of ± 9 ISS points, we did not observe a good precision of the sAIS to calculate the ISS. The AIS was conceived to standardize the classification of traumatic injuries. Since 1971 and with each update and revision, the catalogue has evolved by incorporating new descriptors, refining existing ones and introducing specific coding rules [13, 14]. These enhancements have not only augmented its completeness, but also increased its complexity. The development of the sAIS was driven by the need of a pragmatic, easy-to-use classification, centred on the most frequently reported injuries. In 1998, a first attempt was proposed by Civil et al. who developed a condensed chart (CAIS-85) for the clinical use of the AIS ©1985, but unfortunately its performance was not assessed [15]. To our knowledge, no study has assessed a simplified or a condensed chart of the AIS in multiple trauma. Only one Brazilian study assessed the CAIS-85 in head injury. They found a similar ISS calculated with the CAIS-85 compared with the reference method with AIS/90 [16].

Our findings showed that the sAIS allowed to accurately estimate the ISS with an non-significant ISS difference at the population level, particularly in patients with a higher ISS. However, the reference method for ISS calculation is not without limitations. Ringdal et al. showed that even with AIS-certified coders in the Norwegian trauma system, inter-rater agreement was poor [11]. The Dutch system found an inter-rater agreement rate of 49% [17]. Reliability could be improved by a one-day training course at regular intervals by coding meetings or by calibration of cases coded by all coders [18, 19]. The accuracy of the reference method with the complete AIS catalogue was frequently reported as poor. Twiss et al. reported an accuracy of 42% for ISS coding in the Dutch system [17]. In North America, Arabian et al. reported 64% of accuracy for AIS coding by registrars in state-verified level I and II trauma centres [20]. Poor accuracy and reliability of the reference method for AIS coding highlight its complexity. In addition, the limitation of the reference method is likely to create an imperfect gold standard bias [21].

At an individual patient level, this study showed a low precision with a LoA slightly wider than the predefined relevant limit of ± 9 ISS points. The low precision occurred mainly for a higher ISS due to the squaring of each AIS severity code. We observed that the LoA were exceeded for ISS values > 30. At an individual patient level, this is probably less important. Of note, the ISS is useful for benchmarking in trauma audit and research, but not for individual decision-making [22]. It was demonstrated that the ISS is a mathematical function useful to retrospectively assess priority of care, rather than cardinal numbers reflecting the human body response to multiple injuries [23–25]. We recommend scoring the sAIS of all injuries as this approach allows not only the adequate calculation of the sISS, but also the creation of subgroups with specific injury patterns, e.g., all cases with a femur fracture.

### Clinical implications

The sAIS allows to calculate the sISS in institutions without a capacity of trained and certified staff to code the rISS. This issue affects not only low- and middle-income countries (LMICs), which carry the highest burden of injuries and yet struggle to initiate care improvement programmes, but also high-income countries (HICs). In HICs, only the main trauma centres can finance certified coders. Thus, reliable data to estimate the burden of injuries and calculate the ISS for benchmarking are essential, even in smaller hospitals with limited resources that still care for injured patients. A less precise, but more accessible method for ISS coding would facilitate the implementation of quality improvement programmes in settings with a high incidence of traumatic injuries, including audit and benchmarking. However, staff scoring the sAIS still require training to understand medical terminology and accurately extract information from medical records. Notably, a simple and available ISS coding tool could facilitate the inclusion of LMICs in international trauma research collaborations. One of the criticisms of clinical trials of LMICs is the lack of data on patient severity, particularly ISS data [26]. While most severe trauma cases occur in LMICs, the majority of trauma trials were conducted in HICs [27]. Nevertheless, many trials conducted in HICs were underpowered and experienced difficulties in patient recruitment [28, 29]. Inclusion of patients from LMICs could help to conduct large trials, such as the CRASH trials [30].

### Strengths and limitations

A key strength of the study is that we used data collected by robust and established trauma registries. In addition, AIS and ISS coding were performed by AAAM-trained nurses. Despite this, our study has some limitations. As Swiss regulations require written consent for non-interventional studies, we included exclusively patients with written informed consent. This pre-selection of cases may have led to a selection bias in the external validation dataset. However, randomization and stratification on the ISS ensured sufficient representativeness for the purpose of this diagnostic accuracy and validation study, including the use of appropriate statistical methods at the population and individual levels. Of note, an imperfect gold standard bias may have reduced the performance of the sAIS method or simply reproduced the weakness of the reference method. We performed a robust external validation using different study participants and trauma cases and not just a temporal validation like many validation studies [31]. Nevertheless, external validation was based on data collected from a similar population in terms of case-mix as the data used for the sAIS development and internal validation. The time periods for the collection of the two datasets were also different. A study exploring inter-rater reliability will be necessary, as well as an external validation study in settings with a different socio-demographic index and including larger populations.

## Conclusions

This study assessed the accuracy and precision of a new simplified method to quantify the severity of injury using a sAIS classification to calculate the ISS. The tool is accurate, but slightly imprecise in calculating the ISS. On a population level, the accuracy of the ISS difference makes it acceptable for conducting audits of trauma centres and systems. The development of this scale increases the possibilities to use a scoring system for severely injured patients in settings where there is a limited availability of specifically trained and accredited staff.

## Supplementary Information

Additional file 1.

## Data Availability

The datasets used and analyzed during the current study are available from the corresponding author upon request.

## Abbreviations

AAAM: Association for the advancement of automotive medicine
AIS: Abbreviated injury scale
BP: Blood pressure
CI: Confidence interval
ED: Emergency department
GCS: Glasgow coma scale
HICs: High-income countries
ICU: Intensive care unit
IQR: Interquartile range
ISS: Injury severity score
LMICs: Low- and middle-income countries
LoA: Limit of agreement
rAIS: Reference abbreviated injury scale
rISS: Reference injury severity score
sAIS: Simplified abbreviated injury scale
SD: Standard deviation
sISS: Simplified injury severity score
TRAC: Trauma registry of acute care
TRENAU: Northern French Alps trauma registry

## References

[1] Nathens AB, Jurkovich GJ, Rivara FP, Maier RV. Effectiveness of state trauma systems in reducing injury-related mortality: a national evaluation. JTrauma Acute Care Surg. 2000;48:25. 10.1097/00005373-200001000-00005 10647561

[2] Coccolini F, Kluger Y, Moore EE, Maier RV, Coimbra R, Ordoñez C, et al. Trauma quality indicators: internationally approved core factors for trauma management quality evaluation. World J Emerg Surg. 2021;16:6. 33622373 10.1186/s13017-021-00350-7 PMC7901006

[3] Association for the Advancement of Automotive Medicine. Abbreviated Injury Scale (AIS); 2022. https://www.aaam.org/abbreviated-injury-scale-ais/. Accessed 27 Nov 2024.

[4] Baker SP, O’Neill B, Haddon W, Long WB. The injury severity score: a method for describing patients with multiple injuries and evaluating emergency care. J Trauma. 1974;14:187–96. 4814394

[5] Spence RT, Zargaran E, Hameed M, Fong D, Shangguan E, Martinez R, et al. Injury severity score coding: data analyst v. emerging m-health technology. South Afr Med J. 2016;106:1037–41. 10.7196/SAMJ.2016.v106i10.10597 27725025

[6] Bossuyt PM, Reitsma JB, Bruns DE, Gatsonis CA, Glasziou PP, Irwig L, et al. STARD 2015: an updated list of essential items for reporting diagnostic accuracy studies. BMJ. 2015;351: h5527. 26511519 10.1136/bmj.h5527 PMC4623764

[7] Cohen JF, Korevaar DA, Gatsonis CA, Glasziou PP, Hooft L, Moher D, et al. STARD for Abstracts: essential items for reporting diagnostic accuracy studies in journal or conference abstracts. BMJ. 2017;358: j3751. 28819063 10.1136/bmj.j3751

[8] Northern French Alps Emergency Network. Trauma registry. https://www.renau.org/traumatologie-trenau. Accessed 24 Nov 2024.

[9] Registre Trauma CHUV [Trauma registry. Lausanne University Hospital]. https://www.chuv.ch/fr/chirurgie-viscerale/chv-home/professionnels-de-la-sante/filiere-trauma/registre-trauma-chuv. Accessed 24 Nov 2024.

[10] Ringdal KG, Coats TJ, Lefering R, Di Bartolomeo S, Steen PA, Røise O, et al. The utstein template for uniform reporting of data following major trauma: a joint revision by SCANTEM, TARN, DGU-TR and RITG. Scand J Trauma Resusc Emerg Med. 2008;16:7. 18957069 10.1186/1757-7241-16-7 PMC2568949

[11] Ringdal KG, Skaga NO, Hestnes M, Steen PA, Røislien J, Rehn M, et al. Abbreviated Injury Scale: Not a reliable basis for summation of injury severity in trauma facilities? Injury. 2013;44:691–9. 22831922 10.1016/j.injury.2012.06.032

[12] Landis JR, Koch GG. The measurement of observer agreement for categorical data. Biometrics. 1977;33:159–74. 843571

[13] Committee on Medical Aspects of Automotive Saftey. Rating the severity of tissue damage: I. The Abbreviated Scale. JAMA. 1971;215:277–280. 10.1001/jama.1971.03180150059012 5107365

[14] Association for the Advancement of Automotive Medicine. Abbreviated Injury Scale: 2015 Revision 6th ed.. Chicago, IL; 2018. https://www.aaam.org/abbreviated-injury-scale-ais/. Accessed 24 Nov 2024.

[15] Civil ID, Schwab CW. The abbreviated injury scale, 1985 revision: a condensed chart for clinical use. J Trauma. 1988;28:87–90. 3339667 10.1097/00005373-198801000-00012

[16] de Sousa RM, Koizumi MS, Calil AM, Grossi SA. Chaib L [Measurements of the gravity of injuries in patients with head injuries by the AIS/90 manual and the CAIS/85 chart]. Rev Lat Am Enfermagem. 1998;6:41–51. 9592551 10.1590/s0104-11691998000100007

[17] Twiss E, Krijnen P, Schipper I. Accuracy and reliability of injury coding in the national Dutch trauma registry. Int J Qual Health Care. 2021;33:mzab041. 33693687 10.1093/intqhc/mzab041 PMC7948386

[18] Joosse P, de Jongh MAC, van Delft-Schreurs CCHMK, Verhofstad MHJ, Goslings JC. Improving performance and agreement in injury coding using the abbreviated injury scale: a training course helps. Health Inf Manag. 2014;43:17–22. 24948662 10.1177/183335831404300203

[19] Neale R, Rokkas P, McClure RJ. Interrater reliability of injury coding in the Queensland trauma registry. Emerg Med (Fremantle). 2003;15:38–41. 12656785 10.1046/j.1442-2026.2003.00406.x

[20] Arabian SS, Marcus M, Captain K, Pomphrey M, Breeze J, Wolfe J, et al. Variability in interhospital trauma data coding and scoring: a challenge to the accuracy of aggregated trauma registries. J Trauma Acute Care Surg. 2015;79:359–63. 26307866 10.1097/TA.0000000000000788

[21] Valenstein PN. Evaluating diagnostic tests with imperfect standards. Am J Clin Pathol. 1990;93:252–8. 2405632 10.1093/ajcp/93.2.252

[22] Choi J, Vendrow EB, Moor M, Spain DA. Development and validation of a model to quantify injury severity in real time. JAMA Netw Open. 2023;6: e2336196. 37812422 10.1001/jamanetworkopen.2023.36196 PMC10562944

[23] Dehouche N. The injury severity score: an operations perspective. BMC Med Res Methodol. 2022;22:48. 35184741 10.1186/s12874-022-01528-6 PMC8858478

[24] Abback PS, Brouns K, Moyer JD, Holleville M, Hego C, Jeantrelle C, et al. ISS is not an appropriate tool to estimate overtriage. Eur J Trauma Emerg Surg. 2022;48:1061–8. 33725158 10.1007/s00068-021-01637-9

[25] Vassallo J, Fuller G, Smith JE. Relationship between the Injury Severity Score and the need for life-saving interventions in trauma patients in the UK. Emerg Med J. 2020;37:502–7. 32748796 10.1136/emermed-2019-209092

[26] Napolitano LM, Cohen MJ, Cotton BA, Schreiber MA, Moore EE. Tranexamic acid in trauma: How should we use it? J Trauma Acute Care Surg. 2013;74:1575. 23694890 10.1097/TA.0b013e318292cc54

[27] Vos T, Lim SS, Abbafati C, Abbas KM, Abbasi M, Abbasifard M, et al. Global burden of 369 diseases and injuries in 204 countries and territories, 1990–2019: a systematic analysis for the Global Burden of Disease Study 2019. Lancet. 2020;396:1204–22. 33069326 10.1016/S0140-6736(20)30925-9 PMC7567026

[28] Guyette FX, Brown JB, Zenati MS, Early-Young BJ, Adams PW, Eastridge BJ, et al. Tranexamic acid during prehospital transport in patients at risk for hemorrhage after injury: a double-blind, placebo-controlled, randomized clinical trial. JAMA Surg. 2021;156:11–20. 10.1001/jamasurg.2020.4350 PMC7536625 33016996

[29] Ghossein J, Fernando SM, Rochwerg B, Inaba K, Lampron J, Tran A. A systematic review and meta-analysis of sample size methodology for traumatic hemorrhage trials. J Trauma Acute Care Surg. 2023;94:870. 36879398 10.1097/TA.0000000000003944

[30] Roberts I, Shakur-Still H. Decolonising knowledge production: our experience researching tranexamic acid for trauma victims. BMJ. 2022;377: o1338. 35623649 10.1136/bmj.o1338

[31] Steyerberg EW, Harrell FE. Prediction models need appropriate internal, internal–external, and external validation. J Clin Epidemiol. 2016;69:245–7. 25981519 10.1016/j.jclinepi.2015.04.005 PMC5578404

