# Supplementary material for: Identification of major trauma using the simplified abbreviated injury scale to estimate the injury severity score: a diagnostic accuracy and validation study
Source: Scand J Trauma Resusc Emerg Med. 2025 Jan 29;33:13. doi: 10.1186/s13049-025-01320-7 (PMC11776216; doi:10.1186/s13049-025-01320-7)
Supplement: Supplementary file 1 — Additional file 1. [file 13049_2025_1320_MOESM1_ESM.docx]

**SUPPLEMENT**

**Additional File 1.** Most frequently reported diagnoses in the TRENAU Registry.

**Additional File 2.** Data collection sheet.

**Additional File 3.** Calibration plot and Bland-Altmann of the internal validation dataset**.**

**Additional File 1.** Most frequently reported diagnoses in the TRENAU Registry (2013-2014) (13,361 diagnoses in 3,796 injured patients)

|  |  | AIS code 2005 update 2008 | N | Cum. Freq (%) |
| --- | --- | --- | --- | --- |
| 1 | Pulmonary contusion | 44140#.3 | 619 | (4.6) |
| 2 | Skin: abrasion, contusion | 910000.1 | 545 | (8.7) |
| 3 | Lumbar spine fracture | 65061#.# | 449 | (12.1) |
| 4 | Cerebral concussion (brief LOC) | 161002.2 | 432 | (15.3) |
| 5 | Cerebral concussion (no LOC) | 161001.1 | 377 | (18.1) |
| 6 | Subarachnoid hemorrhage | 140647.3 or 140649.4 or 140647.5 | 371 | (20.9) |
| 7 | Petechial intracerebral hemorrhage | 140642.2 | 347 | (23.5) |
| 8 | Thoracic spine fracture | 65041#.2 | 325 | (25.9) |
| 9 | Pelvic ring fracture (stable) | 856100.2 | 304 | (28.2) |
| 10 | Femur shaft fracture | 853221.3 | 288 | (30.4) |
| 11 | Scalp abrasion | 110099.1 | 282 | (32.5) |
| 12 | Basilar skull fracture | 150200.3 | 272 | (34.5) |
| 13 | Rib fractures (>3) | 450203.3 | 272 | (36.6) |
| 14 | Skin, subcutaneous, muscle injury of the lower extremity | 810099.1 | 239 | (38.3) |
| 15 | Subdural hematoma ≤10 mm | 140652.4 | 219 | (40.0) |
| 16 | Clavicle fracture | 750500.2 | 207 | (41.5) |
| 17 | Skin, subcutaneous, muscle injury of the thorax | 410099.1 | 200 | (43.0) |
| 18 | Skin, subcutaneous, muscle injury of the upper extremity | 710099.1 | 197 | (44.5) |
| 19 | Cervical spine fracture | 650216.2 | 178 | (45.8) |
| 20 | Maxilla fracture | 250800.2 | 168 | (47.0) |
| 21 | Pelvic fracture (incomplete disruption of the posterior arch) | 856161.3 or 856152.3 | 166 | (48.3) |
| 22 | Rib fractures (multiple) | 450210.2 | 166 | (49.6) |
| 23 | Tibia fracture (open) | 854001.3 | 161 | (50.8) |
| 24 | Hemothorax | 442200.3 | 161 | (52.0) |
| 25 | Open fracture of the forearm | 751901.2 | 157 | (53.2) |
| 26 | Forearm Fracture NFS | 751900.2 | 153 | (54.3) |
| 27 | Scapula fracture NFS | 750900.2 | 147 | (55.4) |
| 28 | Pneumothorax | 442202.2 | 146 | (56.5) |
| 29 | Sternum fracture | 450804.2 | 143 | (57.6) |
| 30 | Subdural hematoma large | 140446.5 | 125 | (58.5) |
| 31 | Multiple Rib fractures ≥ 3 | 450203.3 | 123 | (59.4) |
| 32 | Superficial Abdominal contusion | 510402.1 | 116 | (60.3) |
| 33 | Nose fracture close | 251000.1 | 111 | (61.1) |
| 34 | Knee contusion | 810402.1 | 108 | (61.9) |
| 35 | Liver contusion | 541810.2 | 107 | (62.7) |
| 36 | Orbit fracture close | 251200.2 | 107 | (63.5) |
| 37 | Retroperitoneal hematoma | 543800.2 | 104 | (64.3) |
| 38 | Lower limb laceration | 810600.1 | 104 | (65.1) |
| 39 | Spinous ligament cervical | 640284.1 | 100 | (65.8) |
| 40 | Scalp laceration | 110600.1 | 98 | (66.6) |
| 41 | Epidural hematoma small | 140632.4 | 97 | (67.3) |
| 42 | Minor heart contusion | 441002.1 | 93 | (68.0) |
| 43 | Collateral ligament knee | 840400.2 | 93 | (68.7) |
| 44 | Rib fracture 1 | 450201.1 | 93 | (69.4) |
| 45 | Shoulder contusion | 710402.1 | 89 | (70.0) |
| 46 | Tibia fracture | 854000.2 | 88 | (70.7) |
| 47 | Cerebrum contusion (<4cm) | 140640.4 | 85 | (71.3) |
| 48 | Metacarpus fracture | 752500.2 | 85 | (72.0) |
| 49 | Calcaneum fracture | 857300.2 | 84 | (72.6) |
| 50 | Elbow contusion | 772099.1 | 80 | (73.2) |
| 51 | Vault fracture closed | 150402.2 | 78 | (73.8) |
| 52 | Vault fracture complex | 150406.4 | 76 | (74.4) |
| 53 | Spleen laceration (no hilar > 3cm) | 544224.3 | 76 | (74.9) |
| 54 | Skin laceration | 910600.1 | 72 | (75.5) |
| 55 | Humerus fracture open | 751262.3 | 70 | (76.0) |
| 56 | Vertebral cervical fracture | 650230.2 | 66 | (76.5) |
| 57 | Axonal injury (LOC >24h) | 161007.5 | 66 | (77.0) |
| 58 | Spleen subcapsular hematoma | 544214.3 | 63 | (77.4) |
| 59 | Major cerebrum contusion (>4cm) | 140648.5 | 61 | (77.9) |
| 60 | Mandible fracture | 250602.1 | 59 | (78.3) |
| 61 | Dental fracture | 251404.1 | 58 | (78.7) |
| 62 | Flail chest | 450212.3 | 58 | (79.2) |
| 63 | Kidney contusion (subcapsular) | 541612.2 | 56 | (79.6) |
|  | Epidural hematoma large (> 50cc) | 140636.5 | 54 | (80.0) |
| 64 | Vertebral lumbar fracture | 650630.2 | 51 | (80.4) |
| 65 | Humerus fracture close | 751251.2 | 49 | (80.8) |
| 66 | Pelvic ring fracture (incomplete disruption posterior arch) | 856161.3 | 48 | (81.1) |
| 67 | Lung laceration | 441414.3 | 46 | (81.5) |
| 68 | Open chest wound | 415000.4 | 46 | (81.8) |
| 69 | Hip fracture | 853111.3 | 45 | (82.2) |
| 70 | Ankle disruption | 840402.2 | 44 | (82.5) |
| 71 | Hemopneumothorax | 442205.3 | 44 | (82.8) |
| 72 | Flail chest bilateral | 450214.5 | 44 | (83.2) |
| 73 | Vertebral thoracic fracture | 650430.2 | 42 | (83.5) |
| 74 | Cerebrum contusion large | 140608.4 | 42 | (83.8) |
| 75 | Upper limb laceration | 710600.1 | 42 | (84.1) |
| 76 | hemo/pneumothorax major | 442206.4 | 41 | (84.4) |
| 77 | Concussion (LOC 1-6hr) | 161006.3 | 41 | (84.7) |
| 78 | Hip contusion | 873099.1 | 40 | (85.0) |
| 79 | Open nose fracture | 251002.2 | 40 | (85.3) |
| 80 | Hip dislocation | 873030.2 | 39 | (85.6) |
| 81 | Abdominal laceration (>20 cm) | 510604.2 | 39 | (85.9) |
| 82 | Lumbar contusion | 510402.1 | 36 | (86.2) |
| 83 | Rib fractures (2) | 450202.2 | 35 | (86.4) |
| 84 | Liver laceration major | 541826.4 | 35 | (86.7) |
| 85 | Laceration elbow | 710600.1 | 35 | (87.0) |
| 86 | Disjunction metacarpus | 772599.1 | 32 | (87.2) |
| 87 | Wrist contusion | 772499.1 | 31 | (87.4) |
| 88 | Shoulder dislocation | 771030.2 | 30 | (87.6) |
| 89 | Eye injury | 240499.1 | 30 | (87.9) |
| 90 | Astragale fracture | 857200.2 | 29 | (88.1) |
| 91 | Lefort II fracture | 250806.2 | 28 | (88.3) |
| 92 | Basilar skull fracture | 150200.3 | 28 | (88.5) |
| 93 | Vault fracture (massive) | 150408.4 | 28 | (88.7) |
| 94 | Spleen laceration | 544226.4 | 28 | (88.9) |
| 95 | Neck contusion | 310402.1 | 28 | (89.1) |
| 96 | Scalp contusion | 110402.1 | 28 | (89.3) |
| 97 | Lefort III fracture | 250808.3 | 27 | (89.5) |
| 98 | Pelvic ring fracture open | 856162.4 | 26 | (89.7) |
| 99 | Liver contusion | 541810.2 | 26 | (89.9) |
| 100 | Fibula fracture | 854441.2 | 26 | (90.1) |

LOC: loss of consciousness.

N.B. The first 100 most frequently reported diagnoses represent 90% in proportion of reporting of all diagnoses (meaning that some diagnoses of the AIS are very rarely reported).

**Additional File 2.** Data collection sheet.

Thank you for participating! Your contribution to scientific research is greatly appreciated. Please review the medical and radiological records for each of the 10 patients. Using the simplified Abbreviated Injury Scale, assign a value (from 1 to 6) for each anatomical region listed in the table. Ensure that you include every diagnosis, even minor ones such as superficial skin abrasions.

|  | | File | | | | | | | | | |
| --- | --- | --- | --- | --- | --- | --- | --- | --- | --- | --- | --- |
|  |  | A | B | C | D | E | F | G | H | I | J |
| Anatomical region | **Head**  **+**  **neck** |  |  |  |  |  |  |  |  |  |  |
|  |  |  |  |  |  |  |  |  |  |  |  |
|  |  |  |  |  |  |  |  |  |  |  |  |
|  |  |  |  |  |  |  |  |  |  |  |  |
|  |  |  |  |  |  |  |  |  |  |  |  |
|  | **Face** |  |  |  |  |  |  |  |  |  |  |
|  |  |  |  |  |  |  |  |  |  |  |  |
|  |  |  |  |  |  |  |  |  |  |  |  |
|  |  |  |  |  |  |  |  |  |  |  |  |
|  |  |  |  |  |  |  |  |  |  |  |  |
|  | **Chest** |  |  |  |  |  |  |  |  |  |  |
|  |  |  |  |  |  |  |  |  |  |  |  |
|  |  |  |  |  |  |  |  |  |  |  |  |
|  |  |  |  |  |  |  |  |  |  |  |  |
|  |  |  |  |  |  |  |  |  |  |  |  |
|  | **Abdomen** |  |  |  |  |  |  |  |  |  |  |
|  |  |  |  |  |  |  |  |  |  |  |  |
|  |  |  |  |  |  |  |  |  |  |  |  |
|  |  |  |  |  |  |  |  |  |  |  |  |
|  |  |  |  |  |  |  |  |  |  |  |  |
|  | **Extremities**  **+**  **pelvis** |  |  |  |  |  |  |  |  |  |  |
|  |  |  |  |  |  |  |  |  |  |  |  |
|  |  |  |  |  |  |  |  |  |  |  |  |
|  |  |  |  |  |  |  |  |  |  |  |  |
|  |  |  |  |  |  |  |  |  |  |  |  |
|  | **External** |  |  |  |  |  |  |  |  |  |  |
|  |  |  |  |  |  |  |  |  |  |  |  |
|  |  |  |  |  |  |  |  |  |  |  |  |
|  |  |  |  |  |  |  |  |  |  |  |  |
|  |  |  |  |  |  |  |  |  |  |  |  |

Injury Severity Score calculation (for each patient)

|  | max AIS | squared max AIS | ISS  (sum of the square of the three most severely affected body regions)  ------------------ |
| --- | --- | --- | --- |
| Head+neck  Face  Chest  Abdomen  Extremities + pelvis  External |  |  |  |
|  |  |  |  |
|  |  |  |  |
|  |  |  |  |
|  |  |  |  |
|  |  |  |  |


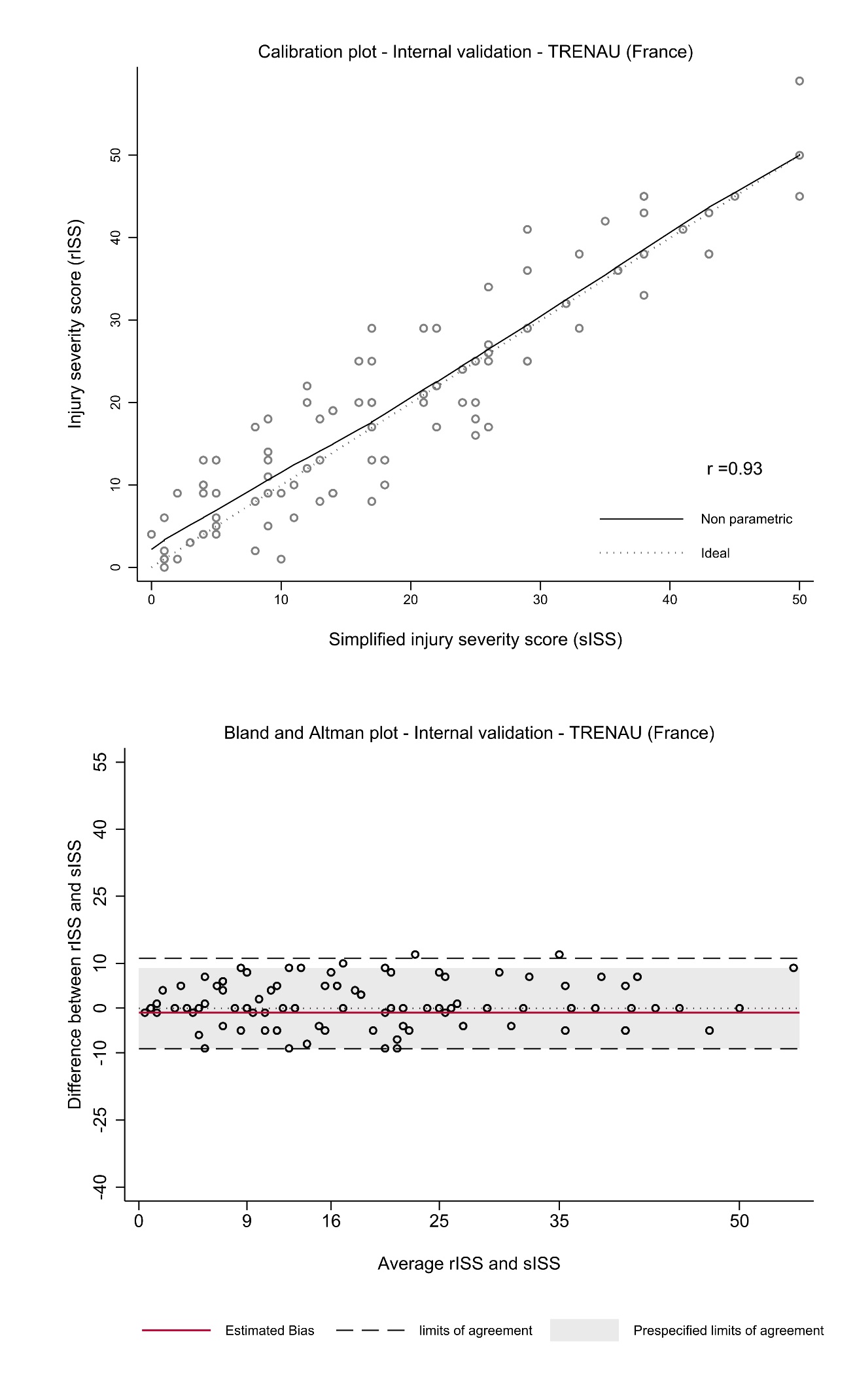
**Additional File 3**: Calibration plot and Bland-Altmann of the internal validation dataset.
